# Empirical mode decomposition processing to improve multifocal-visual-evoked-potential signal analysis in multiple sclerosis

**DOI:** 10.1371/journal.pone.0194964

**Published:** 2018-04-20

**Authors:** Luis de Santiago, Eva Sánchez-Morla, Román Blanco, Juan Manuel Miguel, Carlos Amo, Miguel Ortiz del Castillo, Almudena López, Luciano Boquete

**Affiliations:** 1 Departamento de Electrónica, Escuela Politécnica, Universidad de Alcalá, Alcalá de Henares, Madrid, Spain; 2 Instituto de Investigación Hospital 12 de Octubre (i+12), Madrid, Spain; 3 Departamento de Cirugía, Ciencias Médicas y Sociales, Universidad de Alcalá, Alcalá de Henares, Madrid, Spain; Universita degli Studi di Palermo Dipartimento di Fisica e Chimica, ITALY

## Abstract

**Objective:**

To study the performance of multifocal-visual-evoked-potential (mfVEP) signals filtered using empirical mode decomposition (EMD) in discriminating, based on amplitude, between control and multiple sclerosis (MS) patient groups, and to reduce variability in interocular latency in control subjects.

**Methods:**

MfVEP signals were obtained from controls, clinically definitive MS and MS-risk progression patients (radiologically isolated syndrome (RIS) and clinically isolated syndrome (CIS)). The conventional method of processing mfVEPs consists of using a 1–35 Hz bandpass frequency filter (X_DFT_).

The EMD algorithm was used to decompose the X_DFT_ signals into several intrinsic mode functions (IMFs). This signal processing was assessed by computing the amplitudes and latencies of the X_DFT_ and IMF signals (X_EMD_). The amplitudes from the full visual field and from ring 5 (9.8–15° eccentricity) were studied. The discrimination index was calculated between controls and patients. Interocular latency values were computed from the X_DFT_ and X_EMD_ signals in a control database to study variability.

**Results:**

Using the amplitude of the mfVEP signals filtered with EMD (X_EMD_) obtains higher discrimination index values than the conventional method when control, MS-risk progression (RIS and CIS) and MS subjects are studied. The lowest variability in interocular latency computations from the control patient database was obtained by comparing the X_EMD_ signals with the X_DFT_ signals. Even better results (amplitude discrimination and latency variability) were obtained in ring 5 (9.8–15° eccentricity of the visual field).

**Conclusions:**

Filtering mfVEP signals using the EMD algorithm will result in better identification of subjects at risk of developing MS and better accuracy in latency studies. This could be applied to assess visual cortex activity in MS diagnosis and evolution studies.

## Introduction

### MfVEP

The visual-evoked-potentials (VEP) test is a diagnostic tool that allows an objective assessment of the visual pathway. The conventional visual evoked potential (cVEP) measures the electrophysiological signals obtained by stimulating the full visual field using flash or checkerboard stimuli. The cVEP produces an overall response in the primary visual cortex [1], but it does not provide specific topographical information about the retina and visual cortex [2].

The multifocal-visual-evoked-potentials (mfVEP) technique permits analysis of the topographical features of different sectors of the visual field represented in the visual cortex [3,4]. Several studies [5–7] have shown how the mfVEP technique overcomes most of the limitations of conventional VEPs because it allows for the simultaneous recording of local responses from many visual field sectors (up to 120).

The mfVEP technique has already been shown to be more sensitive than standard automated perimetry for the early detection of visual field defects in multiple sclerosis (MS) [8,9] and other optic neuropathies, such as glaucoma [2].

It is known that the mfVEP signal-to-noise ratio (SNR) is a significant limiting factor for further development of clinical application of mfVEPs, mainly because the amplitudes of the signal recorded are very small compared with cVEP.

In this context, various studies have attempted to improve the diagnostic capacity of the mfVEP technique either by enhancing the visual stimulus parameters [10], by altering the number of electrodes used and their localization, by adding virtual channels [11], by investigating offline signal processing methods such as principal component analysis [12], by using wavelets [13] or by applying Prony’s method as a filter [14].

### MfVEP analysis

Amplitude and latency are the mfVEP signal parameters most frequently used in clinical analysis. MfVEP signal amplitude can be calculated using the difference between the positive peak and the negative trough (peak-to-trough or P2T). P2T has the advantage of being a quantifiable output value (measured in volts) [13,15]. P2T amplitude decline is proportional to the risk of MS [16]. Moreover, an inverse relationship was found between retinal nerve fiber layer thickness—as measured by optical coherence topography—and P2T [15].

MfVEP latency is measured as the delayed conduction of the visual stimulus from the moment it is presented on the screen to the instant it is elicited in the visual cortex. Interocular (IO) latency is defined as the difference between the response latencies of both eyes and is measured as the subtraction between the second-highest peak implicit time [17] or as the temporal shift producing the best cross-correlation value [18].

Delays in mfVEP latency signals are usually observed after an optic neuritis (ON) episode, reflecting optic nerve fiber demyelination, whereas shortening of the latency represents remyelination processes [19,20]. Interocular latency measurements are especially useful in the case of unilateral ON because a normal contralateral eye serves as a good control reference.

### Empirical mode decomposition

Empirical mode decomposition (EMD) has been proposed [21] as an adaptive time–frequency data analysis method. This method decomposes a signal into a sum of oscillatory modes (IMF_1_, IMF_2_, …), called intrinsic mode functions (IMFs), which represent fast to slow oscillations in the signal.

The method successively obtains the highest frequencies (IMF_1_, IMF_2_, …) from a signal, so it is equivalent to a bank of filters of overlapping frequency content. In the electroencephalography (EEG) field, in reference [22] the authors demonstrated that IMF_1_ represents the gamma band neuronal oscillation (>30 Hz), IMF_2_ represents beta band oscillation (13–30 Hz), IMF_3_ represents the alpha band oscillation (8–13 Hz), IMF_4_ represents the delta band (3.5–8 Hz) oscillation, and IMF_5_ and IMF_6_ represent the theta band oscillation (0.5–3.5 Hz).

Typical advanced methods used to process biomedical signals include Fourier [23] and wavelet analysis. These two methods need some predefined basis functions to decompose a signal. In contrast, the EMD method does not require a prior known basis. Comparisons with Fourier and wavelet analyses show that EMD obtains much better temporal and frequency resolutions [24,25].

EMD has been applied to the study of the non-linear and non-stationary properties of time series and has been shown to be a reliable and effective method in the processing of different biomedical engineering signals: enhanced ECG to detect QRS waves [26], detection of components that might be related to phoneme representation in the brain [27], EEG artifact removal [28], and detection and classification of retinal diseases from electroretinogram signals [29]. The authors of [30] used EMD to analyze the neuronal activity of a macaque V4 visual cortex area, showing that evoked potentials can be resolved into a sum of intrinsic components; in a similar experiment, [25] showed that EMD may offer better temporal and frequency resolution in comparison with Fourier analysis. The authors of [31] used EMD to separate EEG components and detect VEPs in EEG signals.

### Aim of this work

The aim of this work was to test, for the first time, application of the EMD preprocessing method to mfVEP signals to improve MS diagnosis.

For this purpose, two experiments were proposed: a) evaluation of the ability to discriminate between mfVEP signals recorded from subjects with different degrees of MS affectation by using the amplitude and b) study of the variability of interocular latency in control subjects.

In the first experiment, we aimed to examine how the amplitude, quantified as P2T, of original and EMD-processed mfVEP signals could be applied to the diagnosis of MS. The difficulty of predicting which patients will develop clinically definite MS currently presents a diagnostic and therapeutic dilemma [32]. This relationship was studied in a cohort of patients with radiologically isolated syndrome (RIS), clinically isolated syndrome (CIS) and clinically definite MS.

In this field, [33] has shown that the mfVEP amplitude, quantified as SNR, performs best at discriminating MS-risk subjects when applied to the visual field (9.8–15° eccentricity, ring 5).

In the second experiment, a comparison of the variability of interocular latency values was made between the mfVEP signals filtered using the standard method and the signals processed using the EMD method.

## Patients and methods

**Fig 1:**
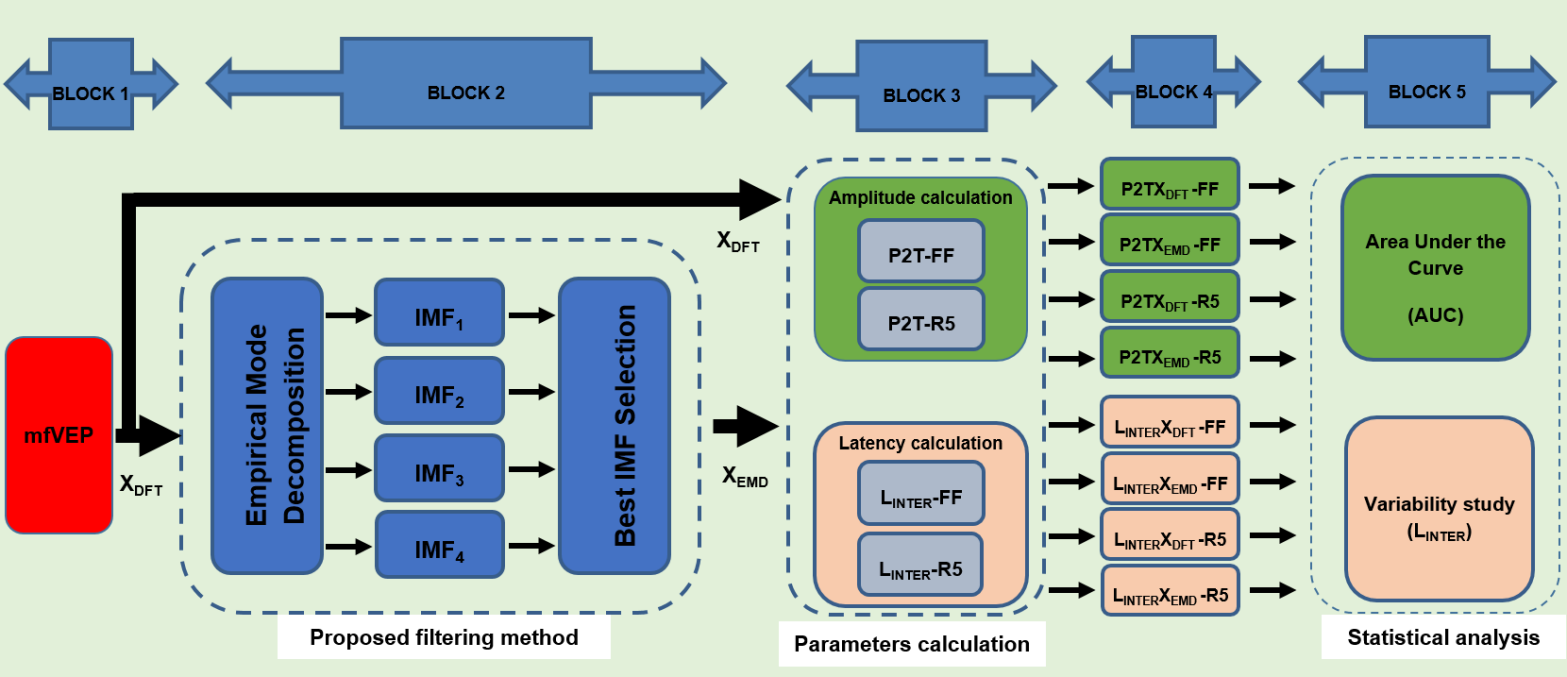
General diagrams of the blocks.

Fig 1 shows a general diagram of the methods used in this research. Briefly, the main blocks are as follows:

BLOCK 1. Represents the typical mfVEP signal-processing method: the mfVEPs of the study participants are recorded, the raw records are digitally filtered by frequency, and the best channel (X_DFT_) for each sector is selected according to the SNR.

BLOCK 2. The EMD decomposition method is applied to X_DFT_ signals in the 45–150 ms interval (signal window).

BLOCK 3. In each sector, only the IMF with the highest amplitude value (quantified as P2T) is selected (X_EMD_) and the other IMFs are discarded.

BLOCK 4. Amplitudes and latencies are computed at several locations in the visual fields (full field and ring 5) of the X_DFT_ and X_EMD_ signals.

BLOCK 5. The discrimination index between controls and patient groups is obtained for the amplitude values. The variability of interocular latency is studied only in the control group. The amplitude and latency results are compared and statistical study is performed.

### Patient database

One cohort of patients with clinically definite MS (n = 28) and two other groups at different relative risks of developing MS—classified as RIS (n = 15) and CIS (n = 28)—were included in this study [34] and compared with a normal, control, age-matched subject group (n = 24) (Table 1).

**Table 1:** Patient demographics.

	Controls	RIS	CIS	MS
Subjects (n)	24	15	28	28
Age (years) mean ± SD	30.3±7.6	39±7.8	30.3±9.6	34.4±10.1
Male:female ratio	10:14	5:10	10:18	7:21
ON-affected eyes	0 (0%)	0 (0%)	12 (21.4%)	37 (66%)
non-ON eyes	48 (100%)	30 (100%)	44 (78.6%)	19 (34%)

RIS subjects are defined as having white-matter anomalies of the central nervous system (CNS)—detected by magnetic resonance imaging (MRI)—that do not account for clinically apparent impairments [35].

CIS subjects are defined as having had a first clinical episode suggestive of CNS demyelination involving the optic nerve, brainstem, spinal cord or other topography not attributable to other inflammatory diseases but lacking radiological evidence of dissemination of lesions over time [36]. It is known that more than 80% of CIS patients who present lesions assessed using MRI eventually develop MS, whereas approximately 20% follow a self-limited process [37].

Clinically definite MS patients were diagnosed according to the McDonald criteria [38].

CIS and MS patients were divided into two subgroups—ON eyes and non-ON eyes—according to whether they had had prior clinical ON episodes.

This study protocol was approved by the Institutional Review Boards of Universidad de Alcalá-affiliated hospitals and adhered to the tenets of the Declaration of Helsinki. All participants provided written informed consent.

### Multifocal visual evoked potentials (mfVEPs)

**Fig 2:**
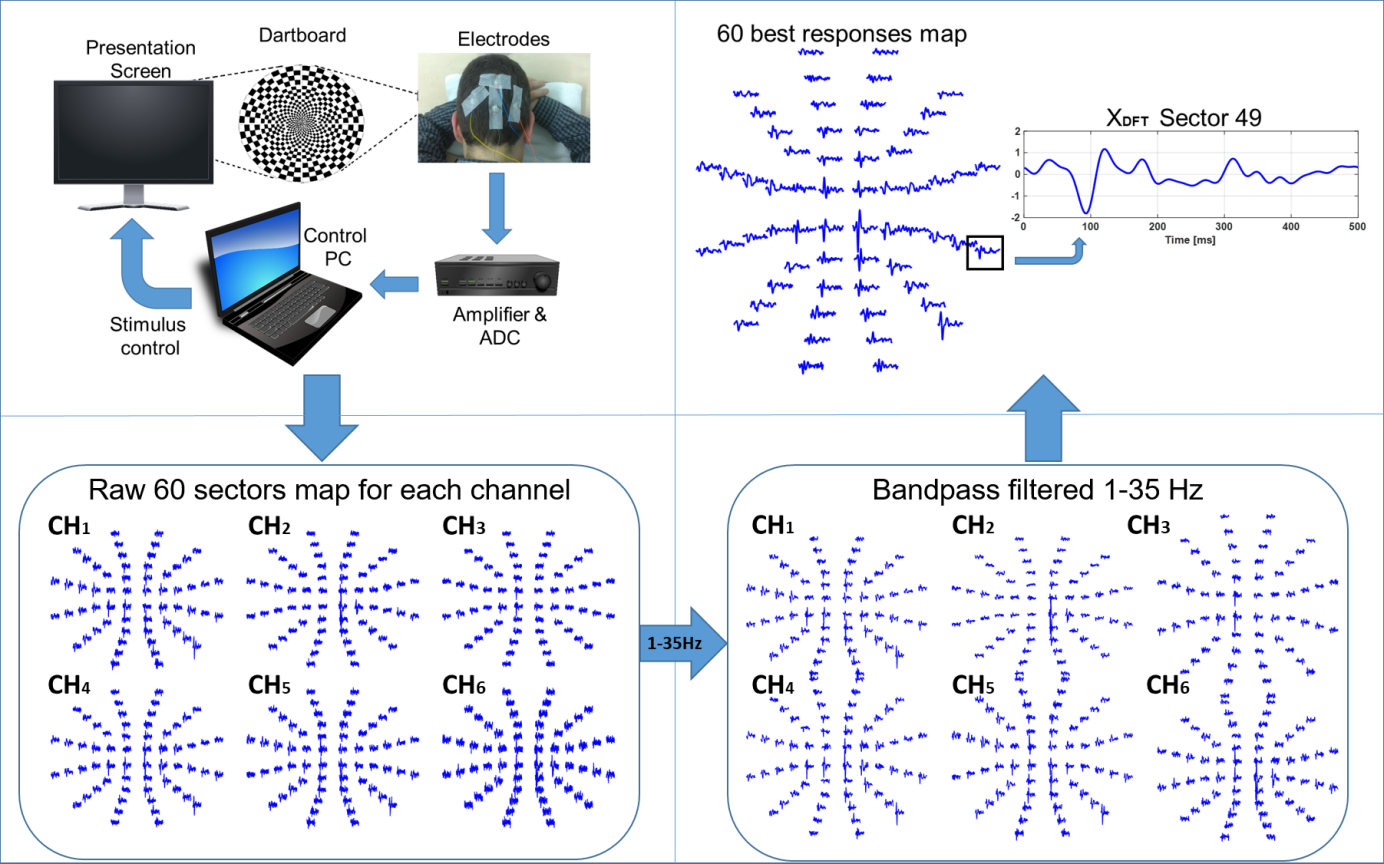
Typical mfVEP analysis method. Upper left: stimulus and recording process; lower left: 60-sector signal map for each of the six channels; lower right: 60-sector signal map for each of the six channels after 1–35Hz bandpass filtering; upper right: 60-sector signal map for the best channel of each sector and signal for one sector.

The typical mfVEP recording and analysis method (Fig 1, BLOCK 1) is represented in detail in Fig 2. Briefly [2,39], mfVEP recordings were obtained using VERIS software 5.9 (Electro-Diagnostic Imaging, San Mateo, USA). The stimulus was a scaled dartboard with a 44.5° diameter containing 60 sectors with 16 alternating checks each—eight white (luminance: 200 cd/m^2^) and eight black (luminance: <3 cd/m^2^)—and a Michelson contrast of approximately 99%. The sectors were cortically scaled with eccentricity to stimulate approximately equal areas of the visual cortex. The dartboard pattern was reversed according to a pseudorandom m-sequence at a frame rate of 75 Hz [2].

Three channels were obtained using gold cup electrodes (impedance <2 kΩ). For the midline channel, the electrodes were placed 4 cm above the inion (active), at the inion (reference), and on the forehead (ground). For the other two channels, the active electrodes were placed 1 cm above and 4 cm on either side of the inion. By taking the difference between pairs of channels, three additional derived channels were obtained, effectively resulting in six channels in each sector (CH_1_ … CH_6_). The length of the recording was 500 ms, and the sample frequency was 1,200 Hz. The signal was analog-amplified (gain: 10^5^, bandwidth: 3–100 Hz) and digital-bandpass-filtered (using a fast Fourier transform: 1–35 Hz).

The recording was divided into two different intervals: the signal window (45–150 ms), which contains the evoked potential response, and the noise window (325–430 ms), which essentially contains noise.

The SNR of each waveform was calculated as SNR = log_10_[RMS(45–150 ms) / mean[RMS(325–430 ms)]], where RMS(45–150 ms) is the root mean square (RMS) amplitude of the waveform in the signal, and mean[RMS(325–430 ms)] is the average RMS amplitude of all 60 waveforms in the noise window (40). The channel with the highest SNR (“best channel”) was selected in each sector (noted as X_DFT_).

### Empirical mode decomposition processing method

Empirical mode decomposition decomposes a non-periodic and non-stationary signal X_DFT_(t) into a finite number of intrinsic mode functions and a residue (Eq 1).

$$X_{DFT}(t)=\sum_{j=1}^{N}{IMF}_{j}(t)+r_{N}(t)$$ (1)

N denotes the total number of IMFs, IMF_j_(t) is the jth IMF and r_N_(t) is the residue selecting N IMFs.

The IMFs must satisfy two main conditions: 1) The number of extremes and the number of zero crossings must be equal or differ by no more than one in the whole dataset; and 2) the mean value of the envelope defined by the local maximum and the envelope defined by the local minimum must be zero at any point (IMFs are nearly periodic functions with zero mean).

First, x(t) = X_DFT_(t) (where x(t) is the input signal) and the IMFs were extracted using the four-step method below:

a) Find all extreme points (maxima and minima) of x(t);
b) Generate the upper and lower envelopes (UE and LE) by interpolation of the maxima and minima with a cubic spline;
c) Compute the mean: $M(t)=\frac{UE(t)+LE(t)}{2}$; and
d) Subtract the mean from the original signal: *c*(*t*) = *x*(*t*) − *M*(*t*).

This process was iterated until the resulting signal c(t) complied with the criteria of an intrinsic mode function. At this point, IMF_1_ = c(t) and the residue r(t) = x(t)-c(t) became the new input signal for step (a) (x(t) = r(t)).

**Fig 3:**
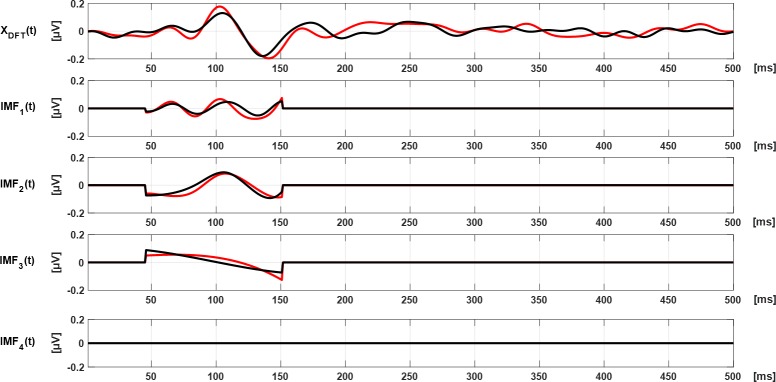
Original signal and IMF_1_–IMF_4_ from the OD (black) and OS (red) from a control subject.

The number of extreme points decreases as the number of previous loop iterations increases. This algorithm stops when r(t) contains one extreme (maxima or minima) or when four IMFs are computed. An example of an mfVEP signal decomposed into IMF_1_–IMF_4_ is shown in Fig 3.

### EMD-based mfVEP filtering method

**Fig 4:**
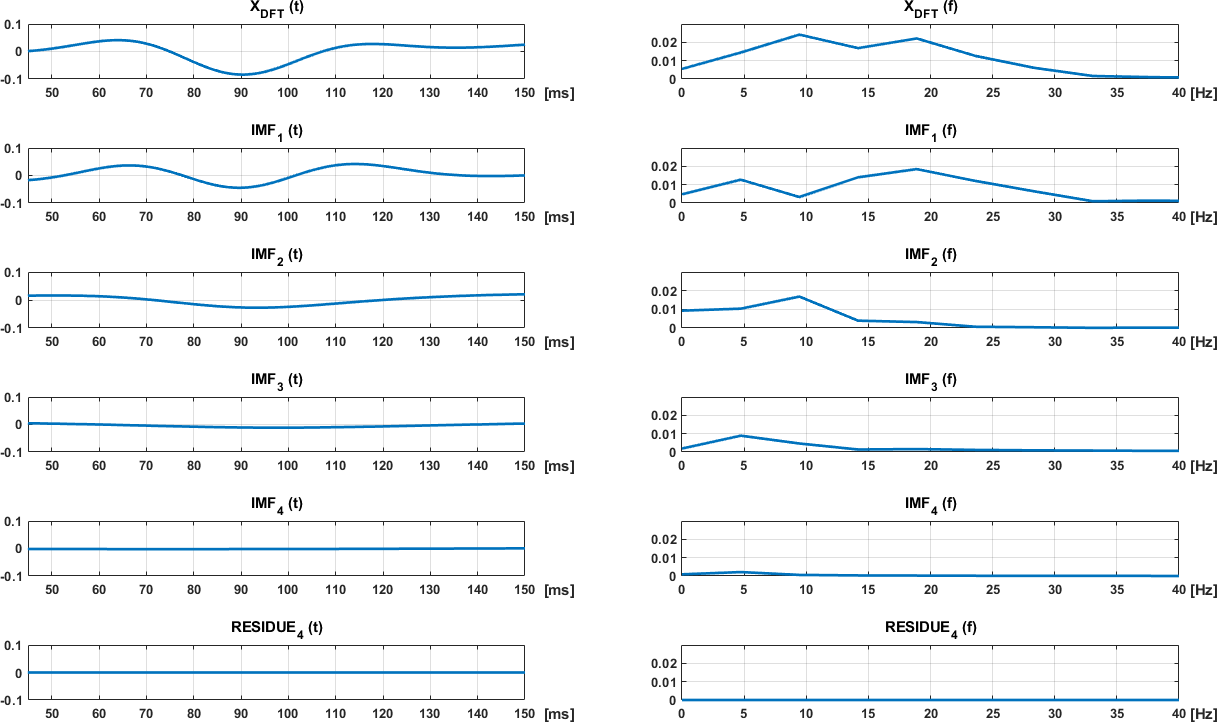
EMD and residues obtained when all the signals in the control database are averaged. Time (left) and frequency (right).

The EMD method is applied to the X_DFT_ signal in the signal window interval X_DFT_(45–150 ms). The number of IMFs computed (N) is selected according to the results shown in Fig 4. This figure shows the IMFs obtained from the average of all the cases in the control database (24 subjects, 2 eyes, 60 sectors, 6 channels). The fourth IMF (IMF_4_) and the residue (*r*_4_(t)) are considered negligible.

Fig 4 also shows each IMF in the frequency domain. As stated in the EMD method, the first IMF is the highest frequency and, as long as the IMF number is increased, the main frequency peak is presented in a lower frequency.

**Fig 5:**
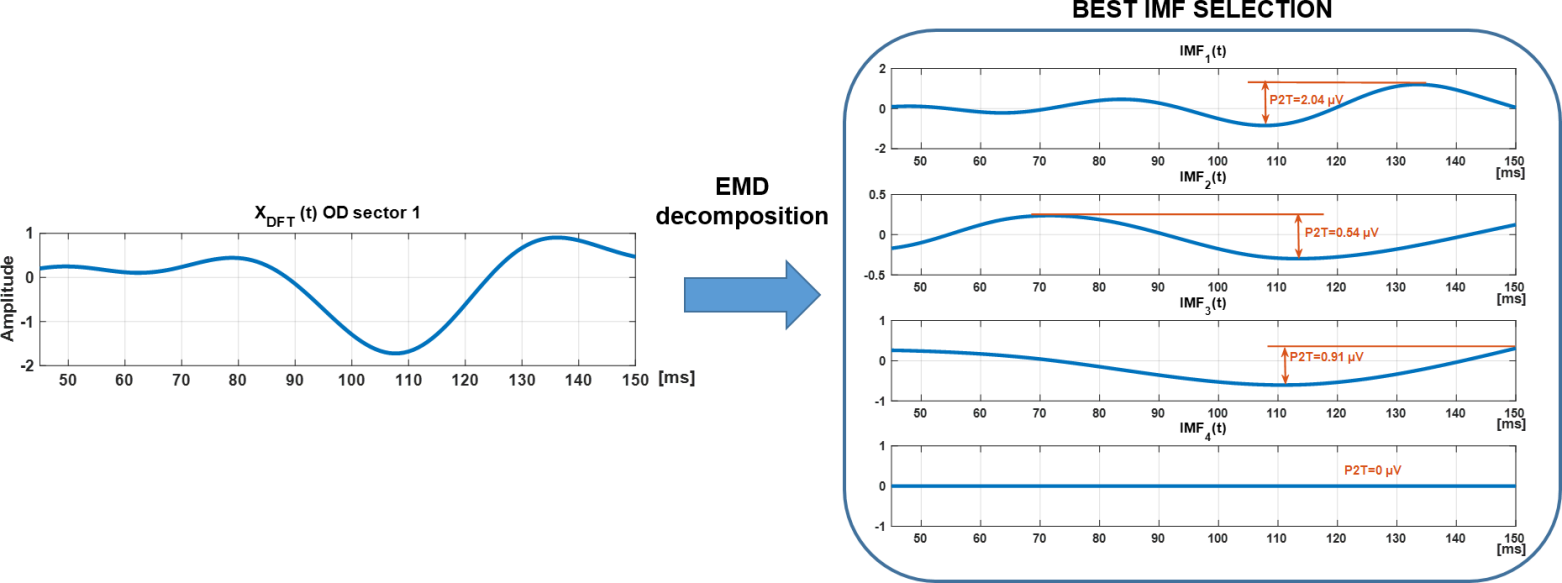
Example of Best IMF selection (OD, sector 1). Highest P2T values is presented in IMF1 so this one is selected as the Best IMF.

EMD is similar to a frequency bank filter. Consequently, there are several filter options depending on which IMFs are selected and which IMFs are discarded. In this paper, only the IMF with the highest P2T amplitude in the signal window is selected (“winner takes all” approach, Fig 5). This IMF (noted as X_EMD_) was considered the filtered signal using the EMD method.

### MfVEP amplitude analysis

**Fig 6:**
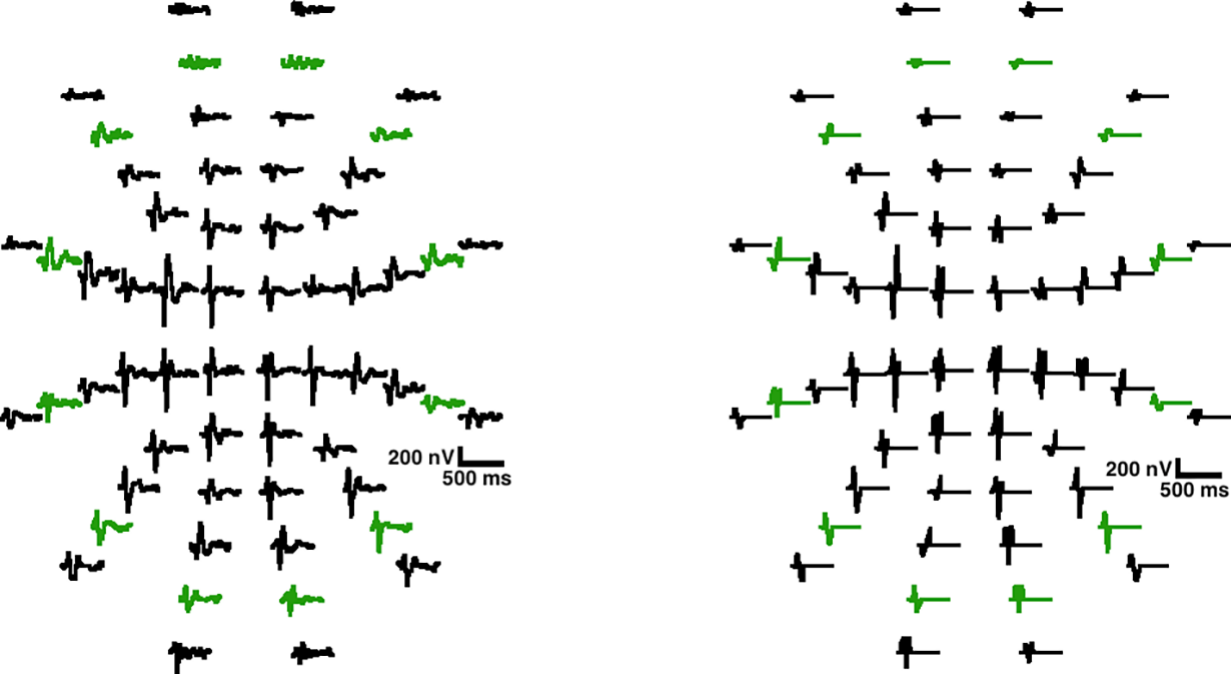
Example X_DFT_ and X_EMD_. Ring 5 signals are colored in green.

The amplitude (P2T) and latency parameters of the signal obtained were computed using the conventional mfVEP analysis method (X_DFT_) and the signals were filtered using EMD (X_EMD_). These parameters were analyzed for both the responses of all 60 recorded sectors (full visual field) and for ring 5 located at 9.8° and 15° eccentricity containing twelve sectors. Fig 6 shows the full field map of X_DFT_ and X_EMD_ signals from a control subject as an example.

MfVEP signal amplitude, quantified as P2T, is the difference between the positive peak and the negative trough in the signal window (45–150 ms). An example is detailed in Fig 5.

### MfVEP latency analysis

**Fig 7:**
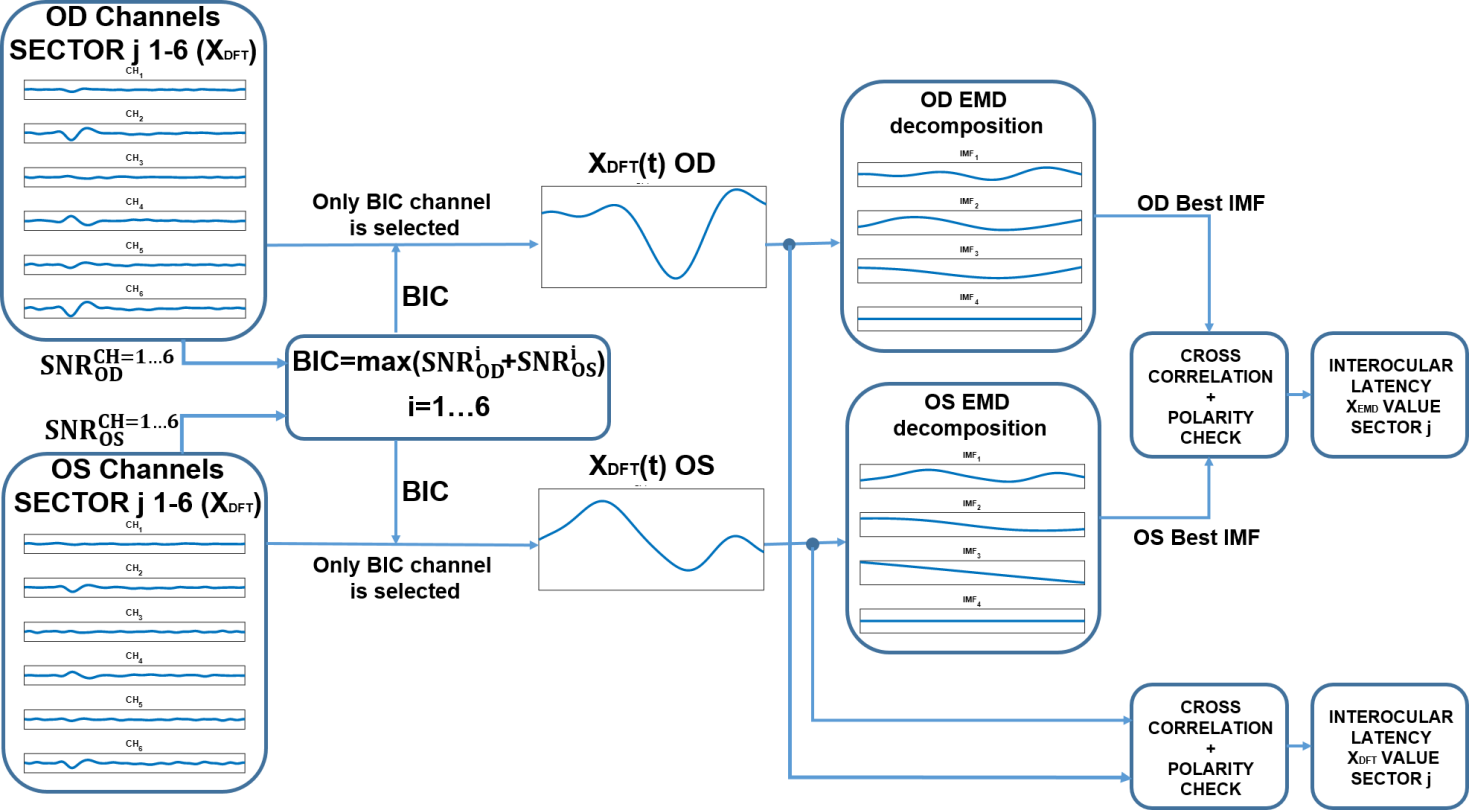
Interocular latency computation flow.

Interocular latency is defined as the mfVEP response delay between both eye signals (L_INTER_ = L_OS_−L_OD_). Measuring the latency comprises the following steps (Fig 7).

a The channel selected in each sector to compute the latency must be the same for both eyes [40]. Thus, the channel that maximizes the sum of SNRs from both eyes is selected (Eq 2) and is noted as best interocular channel (BIC).
  $$BIC=\max\left({SNR}_{OD}^{i}+{SNR}_{OS}^{i}\right),\;i=1\ldots 6$$ (2)
b X_DFT_ signals from the BIC channel of each eye and sector are decomposed into IMFs as previously explained (Fig 1 BLOCK 2 and BLOCK 3).
c The normalized cross-correlations are calculated between the pair of conventional signals (X_DFT-OD_, X_DFT-OS_) and between the pair of IMFs (X_EMD-OD_, X_EMD-OS_). The estimated latency is given by the negative of the lag for which the normalized cross-correlation has the largest absolute value. This step is based on the method described by Hood [41].
d The correlation coefficient between the two signals (OD, OS) is computed to avoid reverse polarity. If this coefficient is negative, the signals are classified as a non-analyzable sector (NAS) and discarded. To obtain the final IO latency for the controls, the IO values of all sectors contained in each zone (full visual field or ring 5) are averaged.

### Variability study (latencies)

Interocular latency should be essentially zero (L_INTER_ ≈ 0 ms) in normal subjects [17,42].

As a statistical measure, intra- and intersubject coefficients of variability were used to compare latencies computed with X_DFT_ and X_EMD_. The intrasubject variability coefficient (CV_INTRA_) was computed (Eq 3) as the mean of the coefficient of variability for each subject (Eq 4). The intersubject variability (CV_INTER_) of latency was calculated as the standard deviation from all subjects’ latency differences divided by the mean latency difference of all subjects (Eq 5).

$${CV}_{INTRA}=Mean({CV\_subject}^{i=1\ldots 24})$$ (3)

$${CV\_subject}^{i}=\frac{{Standard\_Deviation}_{BETWEEN\_ALL\_SECTORS\_SUBJECT\_i}}{{Mean}_{BETWEEN\_ALL\_SECTORS\_SUBJECT\_i}}$$ (4)

$${CV}_{INTER}=\frac{{Standard\_Deviation}_{ALL\;THE\;SUBJECTS\;OF\;THE\;DATABASE}}{{Mean}_{ALL\;THE\;SUBJECTS\;OF\;THE\;DATABASE}}$$ (5)

### Discrimination index (amplitudes)

The discrimination index quantifies the ability to discriminate between controls and patients at different risk of the disease (RIS, CIS and MS). This capacity was evaluated using receiver operating characteristic (ROC) analysis [43]. An ROC curve shows sensitivity against the false positive rate (FPR = 1-specificity) for all possible decision thresholds. The area under the ROC curve (AUC) quantifies the overlaps between P2T value distributions. An AUC value of 0.5 implies that the P2T distributions in both diagnosed groups overlap. AUC values above 0.9 indicate high diagnostic accuracy [44]. AUC values are obtained for X_DFT_ and X_EMD_ signal amplitudes in the full visual field and in the ring 5 eccentricity.

### Statistical analysis

The statistical analysis and study design were based on published recommendations for ophthalmological research [45,46]. The SPSS Version 13 software application (SPSS Inc., Chicago, IL, USA) was used to perform statistical analysis.

The Kolmogorov–Smirnov test was used to determine whether the distribution was normal. A two-sample Student’s t-test was used to evaluate whether the means of two populations were significantly different. The unpaired t-test was used when the two populations were independent, and the paired t-test was used when each value in a group corresponded directly to a value in the other group. One-way ANOVA combined with Tukey’s post-hoc analysis were used to find means that were significantly different in comparisons among more than two groups.

## Results

### Best IMF study

IMF waveforms were obtained for the 22 controls (2 eyes, 6 channels and 60 sectors). All the computed IMFs were then averaged and represented in Fig 4. Since it is possible to check that the main components are IMF_1–3_, it makes sense to compute only the first four IMFs and to discard the residue.

Table 2 shows the proportion of time each IMF was selected (%, in controls) as the best IMF along with the spectral averaged maximum peak frequency. IMF_1_ (peak frequency of 18.9 Hz) was consistently the best IMF (63.14%), and IMF_4_ was never found to be the best IMF.

**Table 2:** Percentage of time as best IMF and maximum peak frequency.

	Original	X_EMD_ decomposed signals			
	X_DFT_	IMF_1_	IMF_2_	IMF_3_	IMF_4_
**% best IMF**	—	63.14%	34.73%	2.12%	0.00%
**Max peak (spectrum)**	9.45 Hz	18.9 Hz	9.5 Hz	4.72 Hz	4.72 Hz

### P2T value analysis

Table 3 shows the P2T values obtained after analyzing all mfVEP-aggregated responses (Full Field: FF) and the eccentric ring 5 (R5) sectors).

**Table 3:** P2T measured for each study group. The values are shown as the mean ± SD.

AMPLITUDE [μV]		CONTROL	RIS	CIS		MS		Mean
				ON	non-ON	ON	non-ON	
**FF**	ORIGINAL X_DFT_	0.66±0.11	0.56±0.10	0.42±0.11	0.55±0.09	0.46±0.11	0.48±0.14	0.55±0.13
	BEST IMF X_EMD_	0.62±0.10	0.48±0.11	0.34±0.09	0.48±0.08	0.41±0.10	0.44±0.13	0.48±0.15
**R5**	ORIGINAL X_DFT_	0.59±0.11	0.37±0.10	0.20±0.06	0.28±0.08	0.22±0.07	0.24±0.09	0.34±0.16
	BEST IMF X_EMD_	0.53±0.10	0.35±0.09	0.19±0.06	0.26±0.07	0.21±0.07	0.22±0.09	0.30±0.30
**Mean**		0.59±0.13	0.41±0.14	0.27±0.14	0.38±0.15	0.31±0.14	0.33±0.16	—

The means were computed using the values from all eyes in the respective group (it is not a mean of means).

A higher significant amplitude (p<0.05) was obtained from the control subjects (0.59±0.13) than from the patients—RIS (0.41±0.14), CIS (ON = 0.27±0.14, non-ON = 0.38±0.15) and MS (ON = 0.31±0.14, non-ON = 0.33±0.16) groups—in the mean value of the four methods analyzed: FF_ORIGINAL_, FF_BEST_IMF_, R5_ORIGINAL_ and R5_BEST_IMF_.

Significantly lower values (p<0.05) were obtained when the best IMF was used in the FF (FF_ORIGINAL_ = 0.55±0.13 > FF_BEST_IMF_ = 0.48±0.15) and R5 (R5_ORIGINAL_ = 0.34±0.16 > R5_BEST_IMF_ = 0.30±0.30) owing to the IMF extraction method.

P2T values were significantly lower (p <0.05) in ring 5 than the full-field aggregated mfVEP responses for both original signals (FF_ORIGINAL_ = 0.55±0.13 vs. R5_ORIGINAL_ = 0.34±0.16) and the best IMF (FF_ORIGINAL_ = 0.48±0.15 vs. R5_ORIGINAL_ = 0.30±0.30).

There were no significant differences (p> 0.05) among the mean values for CIS-ON (0.27±0.14), MS-ON (0.31±0.14) and MS-non-ON (0.33±0.16) obtained using the four methods analyzed.

### Discrimination index

Table 4 shows the AUC values, among all study cohorts, when the X_DFT_ signal and best IMF were used. The AUC was computed between the control and the groups of patients. As a general rule, the AUC index increased with higher MS risk. The highest mean discrimination capacity was seen between the control and the MS-ON group $\bar{(AUC}=0.95)$. The lowest mean was seen for RIS patients $\bar{(AUC}=0.71)$, (p<0.05).

**Table 4:** AUC values for RIS, CIS and MS groups.

AUC values		RIS	CIS		MS		Mean
			ON	non_ON	ON	non_ON	
**FF**	ORIGINAL X_DFT_	0.66	0.91	0.84	0.94	0.86	0.84
	BEST IMF X_EMD_	0.68	0.92	0.86	0.95	0.90	0.86
**R5**	ORIGINAL X_DFT_	0.73	0.95	0.92	0.94	0.92	0.89
	BEST IMF X_EMD_	0.76	0.98	0.94	0.97	0.95	0.92
**Mean**		0.71	0.89	0.89	0.95	0.91	—

If the results are compared by method, for all cases the AUC values were higher when X_EMD_ signals were used. The AUC values obtained in ring 5 were higher than the full field values for all cases. The highest mean discrimination capacity was seen in ring 5 when the X_EMD_ signals were used $\bar{(AUC}=0.92)$, (p<0.05).

### Interocular latencies

Table 5 shows the IO latency results obtained for the control cohort: the mean interocular value (L_INTER_), the NAS and the inter- and intrasubject variability.

**Table 5:** L_INTER_: Mean interocular latency values for control patients; NAS: Percentage of non-analyzable sectors; CV_INTER_: Intersubject coefficient of variability; CV_INTRA_: Intrasubject coefficient of variability; FF: Full field; R5: Ring 5.

Latency		L_INTER_ (ms)	NAS %	CV_INTER_	CV_INTRA_
**FF**	Standard X_DFT_	-0.52	9.62	4.66	50.64
	BEST IMF X_EMD_	-0.42	10.76	2.29	42.67
**R5**	Standard X_DFT_	0.29	14.02	3.05	40.54
	BEST IMF X_EMD_	0.24	14.77	1.9	35.74

All mean ***L***_***INTER***_ s are very close to 0 ms, with the value computed using IMF signals in ring 5 (L_INTER_ = 0.24 ms) being the closest. No significant differences were found among L_INTER_ values (p>0.05) between methods and zones.

The standard method applied to the full field showed the lowest number of non-analyzable sectors (9.62%) because this case presents the highest SNR values.

X_EMD_ in ring 5 showed the lowest values regarding intersubject (CV_INTER_ = 1.9) and intrasubject (CV_INTRA_ = 35.74) variability.

For the intersubject CV, no statistical study was performed because, as it is merely a ratio between the standard deviation and the mean of the entire database, only one value was computed ((5).

Significant differences (p<0.05) between zones (full field vs. ring 5) and methods (X_DFT_ vs. X_EMD_) were found for the intrasubject coefficient of variability.

## Discussion

The present study demonstrates that filtering mfVEP signals using the EMD method improves the data-analysis process when applied to diagnosis of MS. Using the amplitude of mfVEP signals (quantified as P2T) filtered with EMD obtains higher discrimination index values than using the conventional method when control, MS-risk progression (RIS and CIS) and MS subjects are studied. Moreover, the interocular latency computation method (cross-correlation [18]) obtains more reliable values if the signals were previously filtered using EMD.

The EMD method decomposes a signal into several IMFs ordered by frequency from high to low. In the mfVEP technique, since the evoked response is presented in the signal window (45–150 ms), the IMF with the highest amplitude value (P2T) in this interval is selected. We believe that the good results presented in this paper derive from the fact that the performance of EMD is similar to that of a bank filter, where each IMF is bandwidth-limited and can be identified as one of the frequency bands of the signal. Since mfVEP components have a low voltage, they are obscured by EEG background activity, making it necessary to define a robust method to extract bands that best describe these potentials of interest (31).

The results from control subjects presented in Table *2* demonstrate that mfVEP signals can be approximated to the first four IMFs. The IMF with the highest P2T value in the signal window is selected as the best IMF. The first IMF (IMF_1_) is selected as the best IMF in 63.14% of the cases and presents a peak amplitude of 18.9 Hz in the averaged spectrum. IMF_2_ is selected as the best IMF in 34.73% of the cases and presents a peak amplitude of 9.5 Hz in the averaged spectrum.

The amplitude P2T values and trends agree with previous papers [16]. These authors have shown that non-ON eye amplitudes of patients with unilateral ON were significantly lower in both CIS and MS when compared with the control.

The mean results (X_DFT_, X_EMD_, both in ring 5 and the full field) also demonstrated this tendency: P2T__CONTROL_ = 0.59±0.13, P2T__CIS-non-ON_ = 0.38±0.15 and P2T__MS-non-ON_ = 0.33±0.16, p<0.05. No significant differences were observed among MS-ON, MS-non-ON and CIS-ON eyes because most non-ON eyes have been shown to be subclinically affected in CIS and clinically definite MS [32].

The lowest P2T values were obtained in CIS-ON (0.27±0.14, p<0.05) eyes because they had a recent ON episode and consequently the amplitudes were still low and recovering. Thus, amplitude P2T values were able to discriminate (p<0.05) CIS-ON (0.27±0.14) eyes from CIS-non-ON eyes (0.38±0.15).

A higher discrimination index (AUC) was achieved when signals filtered with EMD were used when compared with non-EMD-filtered signals (Table 4). The higher the risk of suffering MS, the higher the discrimination index obtained: $\left(\bar{{AUC}_{RIS}}=0.71)<(\bar{{AUC}_{CIS\_ON}}=0.89)<(\bar{{AUC}_{MS\_ON}}=0.95\right)$.

The best mean discrimination index was obtained when using IMFs on ring 5 sectors $\left(\bar{{AUC}_{IMF\;R5}}=0.92\right)$, significantly improving the SNR analysis obtained in our previous work $\left(\bar{{AUC}_{SNR\;R5}}=0.89\right)$ [33]. The main difference is that in our previous work the AUC values were computed using the typical SNR parameter. The innovation in this new paper is the use of empirical mode decomposition.

Other SNR analyses [18,39] have been tested in MS-ON subjects and have previously obtained lower ROC curve AUC values (between 0.86 and 0.91) when compared with our results (MS-ON AUC_IMF_R5_ = 0.97). Those values were aggregated manually in clusters, making them somewhat unreliable. In contrast, we have used an IMF method that is fully automated, which is one reason it was possible to improve the sensitivity of the analysis.

High discrimination index values for CIS-ON (AUC_IMF_R5_ = 0.98) and CIS-non-ON (AUC_IMF_R5_ = 0.94) were obtained using IMF values from ring 5. In the case of the RIS cohort, the discrimination index was not high (AUC_IMF_R5_ = 0.71), implying that P2T may not be sufficient to discriminate RIS.

In summary, the differences in intensity were magnified when IMF values were used to compare all MS-risk groups, making this analysis potentially useful for predicting MS progression.

The second objective of this paper was to improve interocular latency measurements in control subjects by filtering mfVEP signals using EMD. The L_INTER_ latency values obtained were close to 0 ms in all cases. No significant differences were found between the values obtained using the X_EMD_ signals and the standard method.

Variability (CV_INTER_, CV_INTRA_) was reduced in ring 5 compared to the full field. The lowest variability values were found in X_EMD_ signals in ring 5. Significant differences were found in the intrasubject variability between the X_EMD_ and X_DFT_ signals. The lower intrasubject variability obtained with the IMF analysis indicates that the L_INTER_ latency varies very little from sector to sector, so it would be more sensitive in detecting local visual field defects. Moreover, low intersubject variability would simplify the detection of small changes and be especially relevant when comparing subjects at MS risk with visual pathways at different stages of affectation.

The average for the non-analyzable sectors obtained with the EMD analysis was 14.77% (equivalent to 8.62 sectors in a visual field of 60 sectors per eye) compared with 9.62% (equivalent to 5.77 sectors) with X_DFT_ signals. This is because when a different IMF number was selected for each eye as the best IMF, the probability of reverse polarity was increased. Rejecting records from an analysis involves a trade-off between a loss of data on the one hand and a gain in data quality on the other. In this case, we believe that a difference of an average of three sectors is not relevant.

## Conclusions

MfVEP signals filtered using the EMD method improves a) the association of the P2T amplitude values with MS risk and b) the IO latency analysis by reducing variability. Even better results are obtained in ring 5 (9.8–15º eccentricity of the visual field).

The processed signals were selected in two steps: (1) the best channel as a function of the highest SNR and (2) the best IMF as a function of the highest P2T. Thus, the best information is used, thereby improving the results achieved by using IMFs.

These results suggest that mfVEPs can be used to assess visual cortex activity in MS diagnosis and longitudinal studies.

## Supporting information

S1 File Amplitude P2T values and latency interocular values. Parameters measured for each subject of the database.
(DOCX)
