# Correction: Empirical mode decomposition processing to improve multifocal-visual-evoked-potential signal analysis in multiple sclerosis

**DOI:** 10.1371/journal.pone.0196928

**Published:** 2018-05-01

**Authors:** The PLOS ONE Staff

The uploaded S1 File is incorrect. Please view the correct S1 File below. The publisher apologizes for this error.

## Supporting information

S1 File Amplitude P2T values and latency interocular values. Parameters measured for each subject of the database.
(XLSX)

## References

[1] de Santiago L, Sánchez-Morla E, Blanco R, Miguel JM, Amo C, Ortiz del Castillo M, et al (2018) Empirical mode decomposition processing to improve multifocal-visual-evoked-potential signal analysis in multiple sclerosis. PLoS ONE 13(4): e0194964 https://doi.org/10.1371/journal.pone.0194964 29677200 10.1371/journal.pone.0194964 PMC5909914

