# Supplementary material for: Empirical mode decomposition processing to improve multifocal-visual-evoked-potential signal analysis in multiple sclerosis
Source: PLoS One. 2018 Apr 20;13(4):e0194964. doi: 10.1371/journal.pone.0194964 (PMC5909914; doi:10.1371/journal.pone.0194964)
Supplement: S1 File — Parameters measured for each subject of the database. (DOCX) [file pone.0194964.s001.docx]

PONE-D-17-01310R1

Empirical mode decomposition processing to improve multifocal-visual-evoked-potential signal analysis in multiple sclerosis

Dear Dr. Boquete:

Thank you for returning your manuscript and for your continued cooperation with our requests. There are still some outstanding issues that must be resolved before your manuscript can be formally accepted – please see below.

To access your manuscript and complete these changes, please follow this link: https://pone.editorialmanager.com/l.asp?i=29819779&l=5WXWVS13.

You will find the submission in “Current Task Assignments” and will need to click “Submit Task” in order to upload your corrected files. Your current files can be downloaded through the “Assignment Files” link; you can also download the manuscript file attached to this email.

Your task is due Apr 12 2018 11:59PM.

We appreciate your patience during this process. Please contact plosone@plos.org with any questions or concerns.

With kind regards,

PLOS ONE staff

JOURNAL REQUIREMENTS:

1. Thank you for providing your updated Funding Statement. As a general policy, PLOS ONE encourages a complete disclosure of all funders even if a funder no longer contributes to the study. If support was received at one point in time during the experiment, it is important to include it in the financial disclosure nevertheless.

Before we can proceed with production, please explain why the following funder was removed from your Funding Statement and whether you did indeed receive funding from them during the course of your study: Advanced analysis of mfERG and mfVEP applied to the diagnosis of optic neuropathies” (ref. TEC2011-26066)

**Author´s response:**

The inclusion of the funder: Advanced analysis of mfERG and mfVEP applied to the diagnosis of optic neuropathies” (ref. TEC2011-26066) there was an error.

I declare that we have not received funding from TEC2011-26066 for the realization of this paper.

Luciano Boquete
